# Does “One Size Fits All”? Rethinking FIGO Depth of Invasion Measurements in Vulvar Cancer

**DOI:** 10.1097/PGP.0000000000001009

**Published:** 2024-02-02

**Authors:** Maaike C.G. Bleeker, Tjalling Bosse, Koen K. van de Vijver, Joost Bart, Hugo Horlings, Trudy G.N. Jonges, Nicole C.M. Visser, Loes F.S. Kooreman, Johan Bulten, Patricia C. Ewing-Graham

**Affiliations:** Department of Pathology of Amsterdam University Medical Center (M.C.G.B.); Department of Pathology of Netherlands Cancer Institute/Antoni Van Leeuwenhoek Hospital (H.H.), Amsterdam; Department of Pathology of Leiden University Medical Center, Leiden (T.B.); Department of Pathology of University Medical Center Groningen, Groningen (J.B.); Department of Pathology of University Medical Center Utrecht, Utrecht (T.G.N.J.); Department of Pathology of Eurofins PAMM, Eindhoven (N.C.M.V.); Department of Pathology and GROW—School for Oncology and Reproduction, Maastricht University Medical Center, Maastricht (L.F.S.K.); Department of Pathology of Radboud University Medical Center, Nijmegen (J.B.); Department of Pathology of Erasmus Medical Center, Rotterdam (P.C.E.-G.), The Netherlands; Department of Pathology of University Gent, Gent, Belgium (K.K.v.d.V.)

**Keywords:** Vulvar cancer, Depth of invasion, Applicability, FIGO 2009, FIGO 2021

## Abstract

Depth of invasion (DOI) is an important diagnostic parameter in patients with vulvar carcinoma, where a cutoff value of 1 mm largely determines the tumor stage and the need for groin surgery. DOI measurement should be reproducible and straightforward. In light of the new recommendation on how to measure DOI in the International Federation of Gynecology and Obstetrics (FIGO) staging system 2021, an exploratory study was conducted on the current practice of DOI measurement in vulvar cancer. In this study of 26 selected cases, 10 pathologists with high exposure to vulvar cancer cases in daily practice assessed both the conventional (FIGO 2009) and alternative (FIGO 2021) DOI methods for applicability and preference. In this set of cases, the DOI measurement according to FIGO 2009 was generally considered easier to apply than the measurement according to FIGO 2021, with applicability being rated as “easy to reasonable” in 76.9% versus 38.5% of cases, respectively (*P*=0.005). The preferred method was FIGO 2009 or tumor thickness in 14 cases and FIGO 2021 in 6 cases. No invasion was preferred in 1 case. For the remaining 5 cases, half of the pathologists opted for the FIGO 2009 method and half for the FIGO 2021 method. Although the FIGO 2009 method proved to be more readily applicable in most of the cases studied, the method may differ for each case. There may not be a “one size fits all” solution for all cases of vulvar cancer.

Depth of invasion (DOI) is an important diagnostic parameter in patients with vulvar carcinoma, where a cutoff value of 1 mm largely determines the tumor stage in tumors confined to the vulva/perineum^1,2^. Due to the low risk of lymph node metastasis, lymph node staging is omitted in patients with microinvasive carcinoma (DOI ≤1 mm, International Federation of Gynecology and Obstetrics [FIGO] stage IA), while in patients with macroinvasive carcinoma (DOI >1 mm, FIGO stage ≥IB), groin surgery comprising sentinel node procedure and/or inguinofemoral lymphadenectomy is indicated^1–4^.

There are several methods for measuring DOI in vulvar cancer, including the distance between the epithelial-stromal junction of the most superficial adjacent stromal papillae and the deepest point of invasion (conventional method as recommended in FIGO 2009), the distance between the closest dysplastic epithelium and the deepest point of invasion (alternative method as recommended in FIGO 2021), and the distance between the surface of the dysplastic epithelium and the deepest point of invasion (also known as tumor thickness, Fig. 1)^5,6^.

**FIG. 1:**
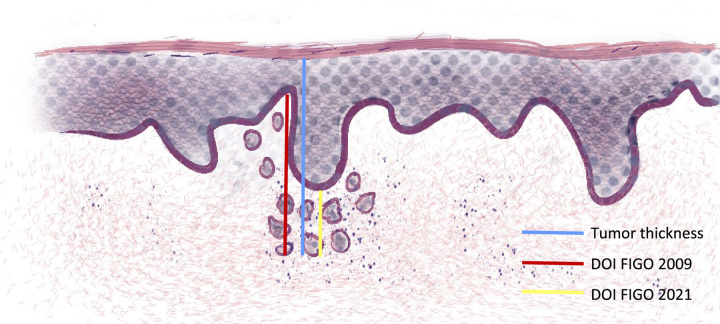
Measurement methods for DOI in vulvar cancer. The conventional method in red (recommended in FIGO 2009) is defined by the distance between the epithelial-stromal junction of the most superficial adjacent stromal papillae and the deepest point of invasion. The alternative method in yellow (recommended in FIGO 2021) is defined by the distance between the closest dysplastic epithelium and the deepest point of invasion. The tumor thickness in blue is defined by the distance between the surface of the dysplastic epithelium and the deepest point of invasion (also known as tumor thickness). DOI indicates depth of invasion; FIGO, International Federation of Gynecology and Obstetrics.

In 2021, the FIGO made several adaptions to the FIGO staging system of vulvar cancer^2^. One important change is an altered recommendation on which method should be used for DOI measurement. The decision to adopt the new DOI measurement method is largely based on a limited number of retrospective studies, suggesting that the alternative method correlates better with clinical outcomes^7,8^. Prospective studies are lacking.

Given the far-reaching consequences of lymph node surgery when the invasion depth is >1 mm, the DOI measurement method used should be readily applicable and reproducible. In practice, the pathologist may be confronted with a variety of histopathologic features, such as growth patterns or ulcerations, which may influence the method of measurement used.

Following the publication of the FIGO staging system 2021, an exploratory study was conducted on the current practice of DOI measurement in vulvar cancer. In this study, pathologists with high exposure to vulvar cancer cases in daily practice assessed both the conventional (FIGO 2009) and alternative (FIGO 2021) DOI methods for applicability and preference.

## MATERIALS AND METHODS

### Selection of Vulvar Cancer Cases

A series of 26 vulvar carcinoma specimens were selected by 3 gynecopathologists (J.B., P.C.E.G., and M.C.G.B.) during their day-to-day work between March 2022 and June 2022 (n=12) or during slide review for other research projects on vulvar cancer (n=14). Cases were selected where there was an expectation that the DOI measurement may change from >1 to ≤1 mm, depending on the method of measurement and/or where DOI measurement was considered challenging. Cases that allowed accurate assessment of invasive tumors relative to the surface were selected; slides with an incomplete tissue section or an ulcerated tumor were not selected. For each case, a digital platform (PathXL; Cirdan Imaging Ltd) was used to annotate the images of the slides with DOI measurements according to distinct methods. For all 26 cases, consensus DOI measurements according to both the conventional method (FIGO 2009) and the alternative method (FIGO 2021) were digitally annotated by the same 3 pathologists. When appropriate, the tumor thickness was also annotated.

### Data Collection

Between June 2022 and August 2022, all slides were assessed independently by 10 pathologists, including 1 pathologist from each gynecologic oncology center in The Netherlands (n=9) and 1 pathologist from a gynecologic oncology center in Belgium. Years of practice as pathologist ranged from 3.7 to 28.7 yr (mean: 14.8 yr). For each case, pathologists assessed the applicability of and preferences for DOI measurement methods using a data collection form. For the DOI according to both FIGO 2009 and FIGO 2021, the following categories were used to record applicability: (i) “easy” (ii) “reasonable” (iii) “moderate” (iv) “difficult” or (v) “not possible.” To evaluate the preferred method of DOI measurement, the following categories were used: (i) tumor thickness, (ii) FIGO 2009, (iii) FIGO 2021, or (iv) no invasion. Pathologists could provide additional comments on the scoring forms. After collecting all data, they were analyzed and discussed during a meeting of the experts in November 2022.

### Statistical Analysis

To assess the applicability of the FIGO 2009 and the FIGO 2021 DOI measurement methods, the categories “easy/reasonable,” “moderate,” or “difficult/not possible” were used. The preferred method was categorized in “Tumor thickness,” “DOI according to FIGO 2009,” “DOI according to FIGO 2021,” or “No invasion.” The agreement for the preferred DOI method per vulvar cancer case was considered “high” when at least 80% of the pathologists preferred the same method, “moderate” when ≥60% to <80% preferred a particular method, and the remaining cases were classed as “no agreement” (<60%). For these calculations, the categories of “*tumor thickness”* and “DOI according to FIGO 2009” were combined, as both methods resulted in very similar measurements (with no cases changing from >1 to ≤1 mm; for details, see Supplementary Table 1, Supplemental Digital Content 1, http://links.lww.com/IJGP/A158). For each case, the scores for the applicability of each DOI measurement method and the preferred measurement method were calculated in proportions. Differences in proportions were calculated using the χ^2^ test. The level of significance was set at *P*-value <0.05.

### Ethics Statement

Anonymized slides were retrieved during daily practice. According to Dutch law, no specific patient approval is required for the use of this material. The study was carried out according to the Code of Proper Secondary Use of Human Tissue (Dutch Federation of Biomedical Scientific Societies, htpp://federa.org).

## RESULTS

### Study Cohort

The characteristics of the 26 vulvar cancer cases are listed in Table 1. The median age of the patients at time of (recurrent) vulvar cancer was 69.2 yr (range: 40–88 yr). The reported FIGO 2009 stages were FIGO IA in 6 (23.1%), FIGO IB in 18 (69.2%), FIGO II in 1 (3.8%), and not available in 1 (3.8%). Vulvar cancer was primary in 22 (84.6%) patients and recurrent in 4 (15.4%) patients. Slides included for DOI assessment of vulvar cancer cases were from resections in 20 (76.9%) and biopsies in 6 (23.1%). Of the 26 selected cases, 20 (76.9%) were human papillomavirus (HPV)-independent and 6 (23.1%) were HPV-associated. In 21 (80.8%) cases, the invasion depth changed from >1 mm (measured according to FIGO 2009) to ≤1 mm (measured according to FIGO 2021). In the other 5 (19.2%) cases, the application of the different measurement methods had no effect on the tumor stage (Supplementary Table 1, Supplemental Digital Content 1, http://links.lww.com/IJGP/A158).

**TABLE 1:** Characteristics of the 26 vulvar cancer cases of the study cohort

Case no.	Age (yr)	Primary or recurrent	FIGO stage	Biopsy or resection	DOI, originally reported	HPV status
1	88	Primary	IB	Resection	1.4 mm	HPV-independent
2	84	Primary	IB	Resection	1.5 mm	HPV-independent
3	78	Primary	IB	Resection	3 mm	HPV-independent
4	76	Primary	IB	Resection	4 mm	HPV-independent
5	69	Primary	IB	Resection	2 mm	HPV-independent
6	88	Primary	IB	Resection	2 mm	HPV-independent
7	72	Primary	IB	Resection	1.5 mm	HPV-independent
8	77	Primary	IB	Resection	1.3 mm	HPV-independent
9	71	Primary	IB	Resection	2 mm	HPV-independent
10	53	Primary	IA	Resection	1 mm	HPV-independent
11	79	Primary	IB	Resection	NR	HPV-independent
12	75	Primary	IB	Resection	NR	HPV-independent
13	78	Primary	IB	Resection	NR	HPV-independent
14	81	Primary	2*	Biopsy	0.9 mm	HPV-associated
15	82	Primary	IB	Biopsy	2.2 mm	HPV-independent
16	52	Primary	IA	Biopsy	0.7 mm	HPV-independent
17	44	Primary	IA	Resection	0.98 mm	HPV-independent
18	40	Primary	IB	Resection	1.4 mm	HPV-associated
19	76	Primary	IA	Resection	Preferably <1 mm	HPV-associated
20	58	Primary	IB	Biopsy	0.8 and 1.7 mm	HPV-associated
21	53	Primary	IB	Biopsy	1.3 and 1.6 mm	HPV-associated
22	52	Recurrent	IA	Resection	<1 mm	HPV-associated
23	50	Recurrent	NR	Resection	1.1 and 1.6 mm	HPV-independent
24	72	Recurrent	IB	Biopsy	2 mm	HPV-independent
25	80	Recurrent	IA	Resection	<0.5 mm	HPV-independent
26	71	Primary	IB	Resection	3 mm	HPV-independent

* Based on anal involvement.

DOI indicates depth of invasion; FIGO, International Federation of Gynecology and Obstetrics; HPV, human papillomavirus; NR, not reported.

### Applicability of DOI Measurement Method

**FIG. 2:**
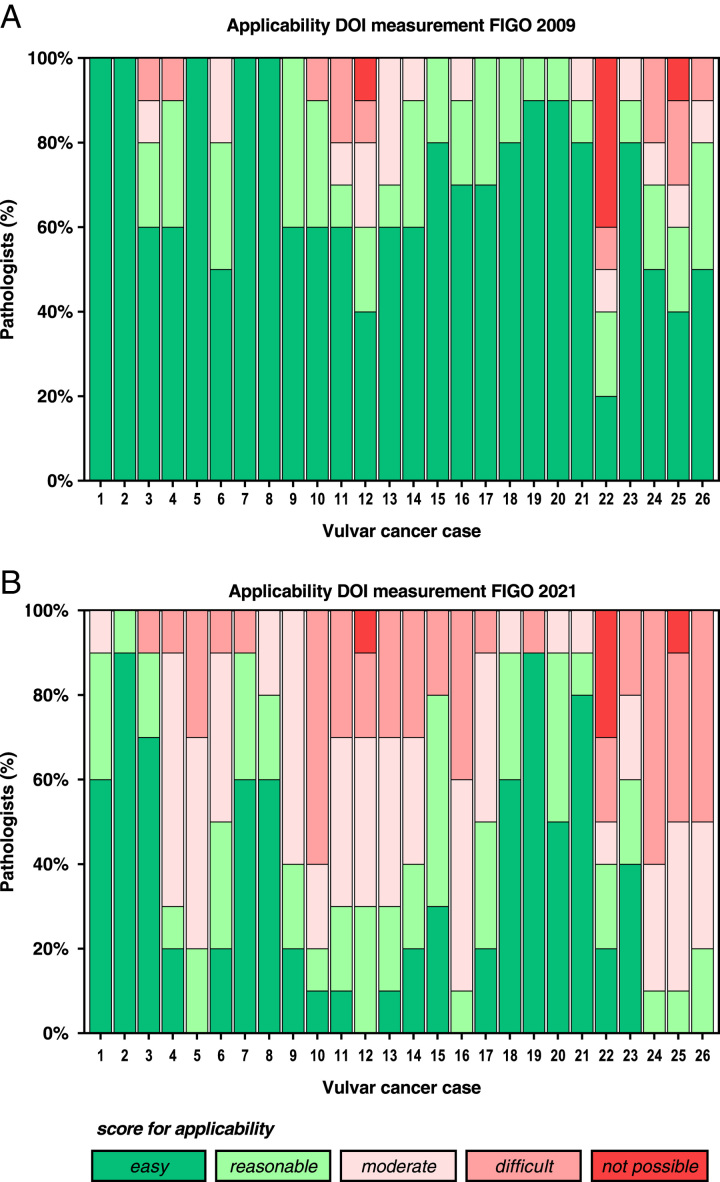
Applicability of FIGO 2009 (A) and FIGO 2021 (B) method for DOI measurement in vulvar cancers. DOI indicates depth of invasion; FIGO, International Federation of Gynecology and Obstetrics.

Applicability was assessed for 26 vulvar cancer cases by 10 pathologists, resulting in 260 assessments per method. The applicability of the conventional (FIGO 2009) and alternative (FIGO 2021) methods for DOI measurement differed per case (Fig. 2). Applying the FIGO 2009 method was found to be “easy/reasonable” in 225 (86.5%) of 260 assessments, “moderate” in 17 (6.5%), and “difficult/not possible” in 18 (6.9%). For FIGO 2021 method, the applicability was considered “easy/reasonable” in 138 (53.0%), “moderate” in 66 (25.4%), and “difficult/not possible” in 56 (21.5%) of 260 assessments. Applicability was significantly better (i.e. easier) for DOI measurements according to FIGO 2009 than FIGO 2021 (*P*<0.0001). When considering the 26 cases studied, the applicability of the method used was perceived as “easy/reasonable” in 20 cases (76.9%) by at least 80% of the pathologists when the FIGO 2009 method was applied, and 10 (38.5%) when the FIGO 2021 method was applied (*P*=0.005). The detailed scores for applicability per method and case are presented in Supplementary Table 1 (Supplemental Digital Content 1, http://links.lww.com/IJGP/A158). For both the FIGO 2009 and the FIGO 2021 method, there was no significant difference in applicability between vulvar resections and biopsy cases nor between HPV-independent and HPV-associated cases (results not shown).

### Preferred Method of DOI Measurement

**FIG. 3:**
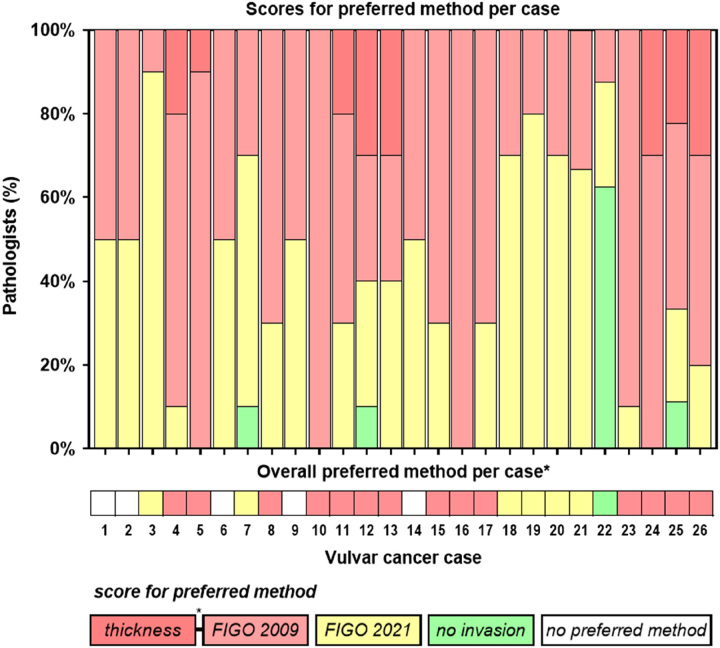
Preferred method for depth of invasion measurement in vulvar cancers. FIGO indicates International Federation of Gynecology and Obstetrics.

The preferred method for measuring DOI, as indicated by 10 pathologists for the 26 cases studied, is presented in Figure 3. The FIGO 2009 method was preferred in 134 (52.3%) assessments, the FIGO 2021 method in 95 (37.1%), and tumor thickness in 19 (7.4%). No invasion was accorded in 8 (3.1%). Data on the preferred method were not provided for 4/260 (1.5%) of the assessments. Regarding the 26 cases studied, a high agreement (≥80%) on the preferred method was present for 9 cases (34.6%; 7 FIGO 2009 or thickness, 2 FIGO 2021), moderate agreement (≥60% to <80%) for 12 cases (46.2%; 7 FIGO 2009 or thickness, 4 FIGO 2021, and 1 no invasion), and no agreement (<60%) for 5 cases (19.2%). The detailed scores of the preferred method for each case are presented in Supplementary Table 1 (Supplemental Digital Content 1, http://links.lww.com/IJGP/A158).

### Expert Meeting

**FIG. 4:**
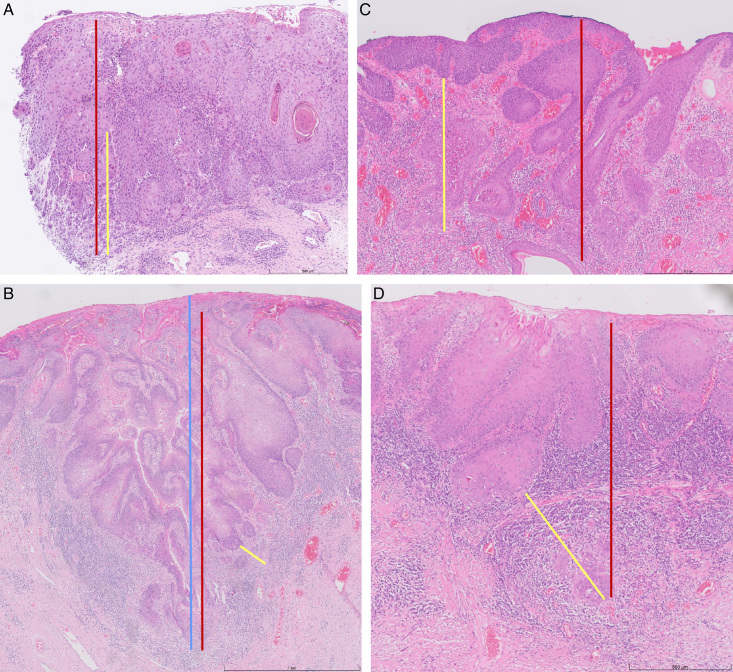
Representative examples of DOI measurements in vulvar cancers. Representative examples of 4 vulvar cancer cases, including the annotations for the DOI measurements according to tumor thickness (blue line), FIGO 2009 (red line), and FIGO 2021 (yellow line). (A) The preferred method for case 16 was FIGO 2009 (preferred by 100% of the pathologists), the applicability of the method to apply was considered “easy/reasonable” by 100% of the pathologists for FIGO 2009 and by 10% of the pathologists for FIGO 2021. (B) The preferred method for case 4 was FIGO 2009 or thickness (preferred by 90% of the pathologists), the applicability of the method to apply was considered “easy/reasonable” by 90% of the pathologists for FIGO 2009 and by 40% of the pathologists for FIGO 2021. (C) The preferred method for case 19 was FIGO 2021 (preferred by 80% of the pathologists), the applicability of the method to apply was considered “easy/reasonable” by 100% of the pathologists for FIGO 2009 and by 90% of the pathologists for FIGO 2021. (D) For case 1, half opted for FIGO 2009 (preferred by 50% of the pathologists) and half opted for FIGO 2021, the applicability of the method to apply was considered “easy/reasonable” by 100% of the pathologists for FIGO 2009 and by 90% of the pathologists for FIGO 2021. DOI indicates depth of invasion; FIGO, International Federation of Gynecology and Obstetrics.

During the expert meeting, the assessments and comments for each case in the study were discussed. There is a consensus that the method of DOI measurement may differ per vulvar cancer case, and this is the approach of most participating pathologists in daily practice. Nevertheless, some cases were very difficult, and uncertainty regarding the best method to be applied remained. Representative examples of vulvar cancer cases including different DOI measurements are shown in Figure 4. Recommendations for the preferred DOI measurement are shown in Table 2 and further discussed in the Discussion section.

**TABLE 2:** Summary and “tips and tricks” for depth of invasion measurement methods

Method of DOI measurement	FIGO 2009	FIGO 2021
Applicability of DOI measurement method	Good	Moderate
Tumor thickness may be an alternative	yes	no
Where there is a limited amount of tumor (“few islands off the coast”) and clear distinction with precursor	no	yes
Where the origin of the tumor is uncertain (“starting point”) and unclear distinction with precursor	yes	no

DOI indicates depth of invasion; FIGO, International Federation of Gynecology and Obstetrics.

## DISCUSSION

In this study, 10 pathologists with a high exposure to vulvar cancer cases in daily practice assessed both the conventional FIGO 2009 and the alternative FIGO 2021 DOI methods for applicability and preference in 26 selected vulvar cancer cases. In this set of cases, the DOI measurements according to FIGO 2009 were considered easier to apply than those of FIGO 2021, with the applicability of the applied method being rated as “easy to reasonable” in 76.9% versus 38.5% of the 26 cases, respectively (*P*=0.005). Of the 26 cases, high agreement on the preferred method to be applied (≥80%) was present in 9 cases (34.6%), moderate agreement (≥60% to <80%) in 12 cases (46.2%), and no agreement (<60%) in 5 cases (19.2%). In the group with moderate to high agreement for the method to be applied, the preferred method was according to FIGO 2009 or thickness in 14 cases and according to FIGO 2021 in 6 cases.

During the expert meeting, the results of the assessments and comments for each vulvar cancer case were discussed. There is consensus that the DOI measurement method to be applied may differ in each case. The recommendations for the preferred DOI method are listed in Table 2. In general, the applicability of DOI measurement according to FIGO 2009 was high. This method was preferred in cases in which the distinction between invasive tumor fields and precursor epithelium was difficult, or where the number of invasive tumor fields was more substantial. DOI measurement according to FIGO 2021 was preferred in cases with a limited number of invasive tumor fields (“a few islands off the coast”) in combination with a well-defined precursor lesion, so that the starting point for the measurement could be clearly defined. In some cases, tumor thickness was preferred, especially in cases with extensive invaginations combined with stromal invasive fields. In line with daily practice, the FIGO 2009 method and tumor thickness resulted in comparable findings, not changing the cutoff of 1 mm.

In <20% of cases, no preferred method was identified, with half of the pathologists opting for the FIGO 2009 method and the other half for the FIGO 2021 method. Our case mix was enriched for challenging cases that impacted the number of vulvar cancer cases for which the choice of the best DOI measurement is not immediately apparent. In an unselected cohort, a lower percentage of disagreement would be expected. It should be noted that, in practice, factors such as ulceration, incomplete sections, or tangential sectioning are more likely to lead to difficulty in DOI measurement than the DOI method to be used^9^.

An important finding of our study was that the applicability of the FIGO 2021 method was perceived as worse than that of the FIGO 2009 method. This could be attributed to many cases in which there was no clear-cut distinction between the precursor lesion and the underlying invasive component. To our knowledge, this is the first study that has examined the applicability of the various methods for measuring DOI. In addition to the method used, the applicability of the DOI measurement may also be related to other factors, such as the type of pathologic specimen. Compared with a resection specimen, it is conceivable that it may be more difficult to find a suitable rete-ridge for DOI measurement in a biopsy specimen. The application of the DOI measurement may also be affected by whether the vulvar cancer is HPV-associated or not. For example, it is possible that the distinction between invasive carcinoma and precursor may be easier in HPV-associated cases than in HPV-independent cases. In our study of 26 selected cases, we found no significant difference in applicability between vulvar resections and biopsy cases, nor between HPV-independent and HPV-associated cases, but our study was not suitable to properly study these factors because both the number of biopsy cases and the number of HPV-associated cases was low.

Consistent with the recently published European Society of Gynaecological Oncology (ESGO) guideline for the management of vulvar cancer^10^, the expert group concluded that there is insufficient evidence to adopt the DOI method according to FIGO 2021 definition as the standard method. Reporting >1 DOI value is not recommended to avoid misunderstandings regarding clinical management. The method of DOI measurement should be reported in the pathology report, particularly when deviating from the conventional FIGO 2009 method. Such and other uncertain cases should be discussed in multidisciplinary team meetings at which the management of oncology patients is discussed.

In a previous study of 148 vulvar carcinomas, the median difference between the FIGO 2009 and FIGO 2021 methods was found to be as much as 1.9 mm, resulting in down staging from IB to IA in 9% of cases^7^. Before guidelines and staging systems adopt the FIGO 2021 method of measurement, more research is needed, ideally with careful reconsideration of which DOI threshold best reflects the clinical outcome. Therefore, prospective studies recording both the FIGO 2009 and the FIGO 2021 method with long-term clinical follow-up data are needed. These studies should include reproducibility of the methods. Although “One size fits all” does not apply to all vulvar cancers, the expert group currently concludes that the FIGO 2009 method can be maintained as the standard for DOI measurements in most vulvar cancers.

## Supplementary Material

SUPPLEMENTARY MATERIAL
