# Supplementary material for: Does “One Size Fits All”? Rethinking FIGO Depth of Invasion Measurements in Vulvar Cancer
Source: Int J Gynecol Pathol. 2024 Feb 2;43(5):457–63. doi: 10.1097/PGP.0000000000001009 (PMC11332368; doi:10.1097/PGP.0000000000001009)
Supplement: SUPPLEMENTARY MATERIAL [file pgp-43-457-s001.docx]

**Table S1**: depth of invasion measurements, applicability and preferred method in 26 vulvar cancer cases

|  | **DOI measurements** | | |  | **Applicability method according to FIGO 2009** | | | | |  |  | **Applicability method according to FIGO 2021** | | | | |  |  | **Preferred method for DOI measurement** | | | |  |
| --- | --- | --- | --- | --- | --- | --- | --- | --- | --- | --- | --- | --- | --- | --- | --- | --- | --- | --- | --- | --- | --- | --- | --- |
| **case** | **Thickness** | **FIGO 2009** | **FIGO 2021** |  | **easy** | **reasonable** | **moderate** | **poor** | **not possible** | **Total** |  | **easy** | **reasonable** | **moderate** | **poor** | **not possible** | **Total** |  | **Thickness** | **FIGO 2009** | **FIGO 2021** | **no invasion** | **Total** |
|  | **mm** | **mm** | **mm** |  | **N *(%)*** | **N *(%)*** | **N *(%)*** | **N *(%)*** | **N *(%)*** | **N *(%)*** |  | **N *(%)*** | **N *(%)*** | **N *(%)*** | **N *(%)*** | **N *(%)*** | **N *(%)*** |  | **N *(%)*** | **N *(%)*** | **N *(%)*** | **N *(%)*** | **N *(%)*** |
|  |  |  |  |  |  |  |  |  |  |  |  |  |  |  |  |  |  |  |  |  |  |  |  |
| **1** | NA | 1.4 | 0.7 |  | 10 *(100)* | 0 *(0)* | 0 *(0)* | 0 *(0)* | 0 *(0)* | 10 *(100)* |  | 6 *(60)* | 3 *(30)* | 1 *(10)* | 0 *(0)* | 0 *(0)* | 10 *(100)* |  | 0 *(0)* | 5 *(50)* | 5 *(50)* | 0 *(0)* | 10 *(100)* |
| **2** | NA | 0.7 | 0.3 |  | 10 *(100)* | 0 *(0)* | 0 *(0)* | 0 *(0)* | 0 *(0)* | 10 *(100)* |  | 9 *(90)* | 1 *(10)* | 0 *(0)* | 0 *(0)* | 0 *(0)* | 10 *(100)* |  | 0 *(0)* | 5 *(50)* | 5 *(50)* | 0 *(0)* | 10 *(100)* |
| **3** | NA | 2.6 | 0.4 |  | 6 *(60)* | 2 *(20)* | 1 *(10)* | 1 *(10)* | 0 *(0)* | 10 *(100)* |  | 7 *(70)* | 2 *(20)* | 0 *(0)* | 1 *(10)* | 0 *(0)* | 10 *(100)* |  | 0 *(0)* | 1 *(10)* | 9 *(90)* | 0 *(0)* | 10 *(100)* |
| **4** | 2.7 | 2.5 | 0.3 |  | 6 *(60)* | 3 *(30)* | 0 *(0)* | 1 *(10)* | 0 *(0)* | 10 *(100)* |  | 2 *(20)* | 1 *(10)* | 6 *(60)* | 1 *(10)* | 0 *(0)* | 10 *(100)* |  | 2 *(20)* | 7 *(70)* | 1 *(10)* | 0 *(0)* | 10 *(100)* |
| **5** | 1.9 | 1.4 | 0.5 |  | 10 *(100)* | 0 *(0)* | 0 *(0)* | 0 *(0)* | 0 *(0)* | 10 *(100)* |  | 0 *(0)* | 2 *(20)* | 5 *(50)* | 3 *(30)* | 0 *(0)* | 10 *(100)* |  | 1 *(10)* | 9 *(90)* | 0 *(0)* | 0 *(0)* | 10 *(100)* |
| **6** | 3.0 | 3.0 | 0.5 |  | 5 *(50)* | 3 *(30)* | 2 *(20)* | 0 *(0)* | 0 *(0)* | 10 *(100)* |  | 2 *(20)* | 3 *(30)* | 4 *(40)* | 1 *(10)* | 0 *(0)* | 10 *(100)* |  | 0 *(0)* | 5 *(50)* | 5 *(50)* | 0 *(0)* | 10 *(100)* |
| **7** | NA | 1.3 | 0.9 |  | 10 *(100)* | 0 *(0)* | 0 *(0)* | 0 *(0)* | 0 *(0)* | 10 *(100)* |  | 6 *(60)* | 3 *(30)* | 0 *(0)* | 1 *(10)* | 0 *(0)* | 10 *(100)* |  | 0 *(0)* | 3 *(30)* | 6 *(60)* | 1 *(10)* | 10 *(100)* |
| **8** | NA | 1.3 | 1.0 |  | 10 *(100)* | 0 *(0)* | 0 *(0)* | 0 *(0)* | 0 *(0)* | 10 *(100)* |  | 6 *(60)* | 2 *(20)* | 2 *(20)* | 0 *(0)* | 0 *(0)* | 10 *(100)* |  | 0 *(0)* | 7 *(70)* | 3 *(30)* | 0 *(0)* | 10 *(100)* |
| **9** | NA | 1.6 | 0.3 |  | 6 *(60)* | 4 *(40)* | 0 *(0)* | 0 *(0)* | 0 *(0)* | 10 *(100)* |  | 2 *(20)* | 2 *(20)* | 6 *(60)* | 0 *(0)* | 0 *(0)* | 10 *(100)* |  | 0 *(0)* | 5 *(50)* | 5 *(50)* | 0 *(0)* | 10 *(100)* |
| **10** | NA | 0.9 | 0.5 |  | 6 *(60)* | 3 *(30)* | 0 *(0)* | 1 *(10)* | 0 *(0)* | 10 *(100)* |  | 1 *(10)* | 1 *(10)* | 2 *(20)* | 6 *(60)* | 0 *(0)* | 10 *(100)* |  | 0 *(0)* | 10 *(100)* | 0 *(0)* | 0 *(0)* | 10 *(100)* |
| **11** | 6.3 | 6.0 | 0.8 |  | 6 *(60)* | 1 *(10)* | 1 *(10)* | 2 *(20)* | 0 *(0)* | 10 *(100)* |  | 1 *(10)* | 2 *(20)* | 4 *(40)* | 3 *(30)* | 0 *(0)* | 10 *(100)* |  | 2 *(20)* | 5 *(50)* | 3 *(30)* | 0 *(0)* | 10 *(100)* |
| **12** | 7.0 | 6.9 | 0.7 |  | 4 *(40)* | 2 *(20)* | 2 *(20)* | 1 *(10)* | 1 *(10)* | 10 *(100)* |  | 0 *(0)* | 3 *(30)* | 4 *(40)* | 2 *(20)* | 1 *(10)* | 10 *(100)* |  | 3 *(30)* | 3 *(30)* | 3 *(30)* | 1 *(10)* | 10 *(100)* |
| **13** | 7.5 | 4.9 | 0.3 |  | 6 *(60)* | 1 *(10)* | 3 *(30)* | 0 *(0)* | 0 *(0)* | 10 *(100)* |  | 1 *(10)* | 2 *(20)* | 4 *(40)* | 3 *(30)* | 0 *(0)* | 10 *(100)* |  | 3 *(30)* | 3 *(30)* | 4 *(40)* | 0 *(0)* | 10 *(100)* |
| **14** | NA | 1.3 | 0.2 |  | 6 *(60)* | 3 *(30)* | 1 *(10)* | 0 *(0)* | 0 *(0)* | 10 *(100)* |  | 2 *(20)* | 2 *(20)* | 3 *(30)* | 3 *(30)* | 0 *(0)* | 10 *(100)* |  | 0 *(0)* | 5 *(50)* | 5 *(50)* | 0 *(0)* | 10 *(100)* |
| **15** | NA | 2.2 | 0.8 |  | 8 *(80)* | 2 *(20)* | 0 *(0)* | 0 *(0)* | 0 *(0)* | 10 *(100)* |  | 3 *(30)* | 5 *(50)* | 0 *(0)* | 2 *(20)* | 0 *(0)* | 10 *(100)* |  | 0 *(0)* | 7 *(70)* | 3 *(30)* | 0 *(0)* | 10 *(100)* |
| **16** | NA | 1.5 | 0.8 |  | 7 *(70)* | 2 *(20)* | 1 *(10)* | 0 *(0)* | 0 *(0)* | 10 *(100)* |  | 0 *(0)* | 1 *(10)* | 5 *(50)* | 4 *(40)* | 0 *(0)* | 10 *(100)* |  | 0 *(0)* | 10 *(100)* | 0 *(0)* | 0 *(0)* | 10 *(100)* |
| **17** | NA | 0.6 | 0.2 |  | 7 *(70)* | 3 *(30)* | 0 *(0)* | 0 *(0)* | 0 *(0)* | 10 *(100)* |  | 2 *(20)* | 3 *(30)* | 4 *(40)* | 1 *(10)* | 0 *(0)* | 10 *(100)* |  | 0 *(0)* | 7 *(70)* | 3 *(30)* | 0 *(0)* | 10 *(100)* |
| **18** | NA | 1.3 | 0.4 |  | 8 *(80)* | 2 *(20)* | 0 *(0)* | 0 *(0)* | 0 *(0)* | 10 *(100)* |  | 6 *(60)* | 3 *(30)* | 1 *(10)* | 0 *(0)* | 0 *(0)* | 10 *(100)* |  | 0 *(0)* | 3 *(30)* | 7 *(70)* | 0 *(0)* | 10 *(100)* |
| **19** | NA | 1.3 | 0.9 |  | 9 *(90)* | 1 *(10)* | 0 *(0)* | 0 *(0)* | 0 *(0)* | 10 *(100)* |  | 9 *(90)* | 0 *(0)* | 0 *(0)* | 1 *(10)* | 0 *(0)* | 10 *(100)* |  | 0 *(0)* | 2 *(20)* | 8 *(80)* | 0 *(0)* | 10 *(100)* |
| **20** | NA | 1.9 | 0.9 |  | 9 *(90)* | 1 *(10)* | 0 *(0)* | 0 *(0)* | 0 *(0)* | 10 *(100)* |  | 5 *(50)* | 4 *(40)* | 1 *(10)* | 0 *(0)* | 0 *(0)* | 10 *(100)* |  | 0 *(0)* | 3 *(30)* | 7 *(70)* | 0 *(0)* | 10 *(100)* |
| **21** | NA | 3.6 | 3.3 |  | 8 *(80)* | 1 *(10)* | 1 *(10)* | 0 *(0)* | 0 *(0)* | 10 *(100)* |  | 8 *(80)* | 1 *(10)* | 1 *(10)* | 0 *(0)* | 0 *(0)* | 10 *(100)* |  | 0 *(0)* | 3 *(33)* | 6 *(67)* | 0 *(0)* | 9 *(100)* |
| **22** | NA | 0.7 | 0.4 |  | 2 *(20)* | 2 *(20)* | 1 *(10)* | 1 *(10)* | 4 *(40)* | 10 *(100)* |  | 2 *(20)* | 2 *(20)* | 1 *(10)* | 2 *(20)* | 3 *(30)* | 10 *(100)* |  | 0 *(0)* | 1 (13) | 2 *(25)* | 5 *(63)* | 8 *(100)* |
| **23** | NA | 1.6 | 0.7 |  | 8 *(80)* | 1 *(10)* | 1 *(10)* | 0 *(0)* | 0 *(0)* | 10 *(100)* |  | 4 *(40)* | 2 *(20)* | 2 *(20)* | 2 *(20)* | 0 *(0)* | 10 *(100)* |  | 0 *(0)* | 9 *(90)* | 1 *(10)* | 0 *(0)* | 10 *(100)* |
| **24** | 2.0 | 1.3 | 1.0 |  | 5 *(50)* | 2 *(20)* | 1 *(10)* | 2 *(20)* | 0 *(0)* | 10 *(100)* |  | 0 *(0)* | 1 *(10)* | 3 *(30)* | 6 *(60)* | 0 *(0)* | 10 *(100)* |  | 3 *(30)* | 7 *(70)* | 0 *(0)* | 0 *(0)* | 10 *(100)* |
| **25** | 1.4 | 1.2 | 0.3 |  | 4 *(40)* | 2 *(20)* | 1 *(10)* | 2 *(20)* | 1 *(10)* | 10 *(100)* |  | 0 *(0)* | 1 *(10)* | 4 *(40)* | 4 *(40)* | 1 *(10)* | 10 *(100)* |  | 2 *(22)* | 4 *(44)* | 2 *(22)* | 1 *(11)* | 9 *(100)* |
| **26** | 3.6 | 3.6 | 0.6 |  | 5 *(50)* | 3 *(30)* | 1 *(10)* | 1 *(10)* | 0 *(0)* | 10 *(100)* |  | 0 *(0)* | 2 *(20)* | 3 *(30)* | 5 *(50)* | 0 *(0)* | 10 *(100)* |  | 3 *(30)* | 5 *(50)* | 2 *(20)* | 0 *(0)* | 10 *(100)* |
|  |  |  |  |  | **181 *(70)*** | **44 *(17)*** | **17 *(7)*** | **12 *(5)*** | **6 *(2)*** | **260 *(100)*** |  | **84 *(32)*** | **54 *(21)*** | **66 *(25)*** | **51 *(20)*** | **5 *(2)*** | **260 *(100)*** |  | **19 *(7)*** | **134 *(52)*** | **95 *(37)*** | **8 *(3)*** | **256 *(100)*** |

*Abbreviations: DOI: depth of invasion; FIGO: International Federation of Gynecology and Obstetrics*
